# Quality and microstructure of freeze-dried yoghurt fortified with additives as protective agents

**DOI:** 10.1016/j.heliyon.2020.e05196

**Published:** 2020-10-09

**Authors:** Elsayed A. Ismail, Ahmed A. Aly, Atallah A. Atallah

**Affiliations:** a Department of Dairy Science, Faculty of Agriculture, Benha University, Benha 13518, Egypt; b Home Economic Department, Faculty of Specific Education, Benha University, Egypt

**Keywords:** Food science, Food analysis, Food technology, Freeze dried yoghurt, Whey proteins, Microstructure, Protective agents

## Abstract

The effect of freeze drying of yoghurt and addition of some additives on the physico-chemical, microbiological, texture, microstructure, and sensory quality of yoghurt was studied. Freeze drying of yoghurt had light effects on the viability of *Streptococcus thermophilus* and no effect on viability of *Lactobacillus delbreuckii* subsp. *bulgaricus* and rehydrated yoghurt. Addition of modified starch had a considerable protection effect on *Streptococcus thermophilus* during freeze drying process. Addition of whey protein concentrate produced different surface structures and caused porous and loos structure. The microstructure of the freeze-dried yoghurt fortified with spirulina powder showed a coarse and a compact less porous structure in comparison with the freeze-dried yoghurt samples fortified with whey protein concentrate. All Additives significantly decreased adhesiveness and significantly increased cohesiveness, springiness, gumminess, and chewiness in both fresh and rehydrated yoghurts.

## 1 Introduction

Yoghurt is a fermented dairy product which is a popular food for many people. Yoghurt contains many nutrients and cultures of *Lactobacillus delbrueckii* subsp. *bulgaricus* and *Streptococcus thermophilus,* which play essential roles in digestion and balancing of micro-flora in the human body (Lopez et al., 1998). Yoghurt and its starter cultures have a limited shelf life in storage. Many ways have been used to extend the yoghurt shelf life such as storage at low temperatures. Freeze drying process is an appropriate method to prolong the shelf life of yoghurt. It includes drying the product through sublimation at low temperatures and under vacuum. This procedure maintains the nutritional, microbiological, and sensory characteristics, and results in a dry product that rehydrates easily (Santos et al., 2018). Jaruwan Chutrtong (2015) reported that freeze-dried yoghurt must be stored in dry conditions at low temperatures. Freeze-dried yoghurt powder may be used as a supplement or an ingredient in some food products (Santos et al., 2018). To enhance the survival of yoghurt traditional cultures (or probiotic microorganisms used for yoghurt manufacturing) during freeze drying process and storage, protective agents should be used (Zayed and Roos, 2004). Carvalho et al. (2002) reported that cryoprotectants such as skim milk or whey protein concentrate are commonly used to protect probiotic cultures during freeze drying. A variety of protectants such as skimmed milk, whey protein, glucose, maltodextrin and trehalose have been used to protect the viability of probiotics during dehydration (Martín et al., 2015). Ainaa et al. (2016) found that the use of guar gum, maltodextrin sucrose during freeze drying enhanced the viability of the freeze-dried yoghurt starter cultures. Akalın et al., (2012) used whey protein concentrates (WPC) to improve the texture and functional properties of yoghurt. Addition of modified starch during yoghurt manufacturing can improve the chemical, sensory and microstructure properties of yoghurt (Khalifa and Ibrahim, 2015). *Spirulina platensis* (blue-green algae) exists in sea water and has high digestibility and has a nutritional resource (Campanella et al., 1998). It contains 70% protein and is a rich source for essential fatty acids, amino acids, minerals, and vitamins, (Mosulishvili et al., 2004). Fortification of yoghurt with spirulina is beneficial in improving the iron content of yoghurt, especially milk is a poor iron source (Malik et al., 2013). The aim of the present study is to prepare freeze-dried yoghurt and investigate its effects with addition of some additives (whey protein concentrate, modified starch, and spirulina powder) on the physico-chemical, microbiological, texture, microstructure and sensory properties.

## 2 Materials and methods

### 2.1 Materials

Whole milk (mixed buffalo and cow's milk 1: 1) was obtained from a farm of the Faculty of Agriculture, Benha University, Egypt. Whey protein concentrate (A253) was obtained from Lacta Company, Poland and modified starch was purchased from Misr for Food Additives (MIFAD) Company, Badr city, Cairo, Egypt. Spirulina (*Spirulina platensis*) powder was obtained through personal communication with a colleague from Arab Academy for Science, Technology, and Maritime transport (AASTMT), Alex., Egypt. Traditional yoghurt starter culture (FD-DVS YC- X11- YO- Flex) containing *Streptococcus thermophilus* and *Lactobacillus delbrueckii* ssp*. bulgaricus* (1:1) was obtained from Chr. Hansen^'^s Laboratories, Copenhagen, Denmark.

### 2.2 Production of yoghurt

Set-type yoghurt was made by using whole buffalo/cow mixed milk (1:1) containing 5% (wt/vol) milk fat. The experiment consisted of 4 treatments as follows: (1) milk was supplemented with 0.5% whey protein concentrate (WPC), (2) milk with 0.5% Spirulina powder (SP), (3) milk with 0.5% modified starch (MS), and (4) milk with no supplements used as control yoghurt (C). Each was heated at 90 °C for 15 min, in a water bath and cooled to 43 °C in a water bath, then 2% of yoghurt starter culture was inoculated. The mixture was poured into plastic containers and immediately incubated at 40 °C for 3–4 h until pH reached to 4.7. Then yoghurt containers were cooled and kept into a refrigerator at 4 °C. The production process of yoghurt was repeated 3 times on different days.

### 2.3 Freeze drying of yoghurt

Freeze drying of yoghurt was carried out using a laboratory freeze dryer (model: VaCo 5-D, S/N: COM98754, Zirbus Technology, Germany). The samples of 300 mL of yoghurt were poured into flasks and frozen to - 20 °C overnight before being transferred to the freeze dryer. Then the samples were transferred to the freeze dryer operating at - 40 °C in a condensation chamber under vacuum at a minimum pressure of 0.011 kPa and maintained during freeze drying. The freeze drying was completed in 72 h, and the resultant flakes were broken and powdered using a mill. The product was kept in sealed polyethylene bags. The yield of freeze-dried yoghurt was calculated as described by Santos et al.(2018). The yoghurt was weighed before and after freeze drying and the yield was calculated according to the following Eq. (1):

(1) $$\text{Yield}\left(\text{\%}\right)=\frac{\text{weight}\;\text{of}\;\text{freeze}\;\text{dried}\;\text{yoghurt}}{\text{weight}\;\text{of}\;\text{traditional}\;\text{yoghurt }}\times 100$$

### 2.4 Rehydration of the freeze-dried yoghurt

Rehydration was carried out as 30g of powder are added to 70g of water. This amount of water represented 82% of water removed from the original yoghurt and this added water was the most appropriate in the pre-experiments.

### 2.5 Physico-chemical properties determinations

Total solids, protein, and ash contents were determined as described by AOAC (2012). The Gerber method (ISO, 2008) was used to determine fat contents. Total carbohydrates were calculated by difference. Titratable acidity was determined according to the method of ISO (2010). The pH was measured using a digital laboratory pH meter (JENCO, model 6173 pH, S/N JC05343, USA).

### 2.6 Texture profile analysis (TPA)

Texture profile analysis was measured using a universal testing machine (TMS-Pro) equipped with 250 lbf load and Texture Pro™ texture analysis software program (DEV TPA). The TPA test set conditions were adjusted to test speed of 60 mm/s, trigger force (1N); deformation (25%) and holding time (2 s) between cycles. Temperature of yoghurt was 5 °C for measuring of TPA. Each sample was subjected to two subsequent cycles (bites) of compression-deformation. Calculations were according to Szczesniak et al. (1963) and Bourne (2002) to obtain the texture profile parameters.

### 2.7 Microbiological analysis

Culture media M17 and MRS agar were used for enumerating *Streptococcus thermophillus* and *Lactobacillus bulgaricus*, respectively. For enumerating of *Streptococcus thermophillus*, the plates were incubated at 37 °C for 48h. For enumeration of *Lactobacillus bulgaricus*, the plates were incubated anaerobically at 37 °C for 72 h (ISO, 2003).

### 2.8 Water holding capacity (WHC)

The WHC was determined according to the method of Akalın et al. (2012) as follows: 20 g of yoghurt were centrifuged for 10 min at 5,000 × *g* and 20 °C. The separated whey was removed and weighed. The WHC was calculated using the following Eq. (2):

(2) $$\text{WHC}\left(\text{\%}\right)=\frac{\text{Yoghurt}\;\text{weight }-\text{Separated}\;\text{Whey}\;\text{weight}}{\text{Yoghurt}\;\text{weight}}\times 100$$

### 2.9 Syneresis index

Syneresis was measured according to the method described by Akalın et al. (2012). A weight of 100g yoghurt was removed and placed into a test sieve (mesh width 0.5 mm). The volume of whey drained off was measured after 120 min at 10 °C. Syneresis was expressed as mL volume of drained whey/100g of yoghurt.

### 2.10 Bulk density of freeze-dried yoghurt powder

Bulk density of freeze-dried yoghurt powder was determined according to the method by Akash and Yugal, (2017) as follows; 25g of powder were taken into a 100mL graduated cylinder, and then the cylinder was shaken by hand 25 times. Final volume rise was measured. The bulk density was expressed as g/mL and calculated according to the following Eq. (3):

(3) Bulk density = powder sample in gram / rise in volume

### 2.11 Solubility index of freeze-dried yoghurt powder

Powder solubility was determined according to the method of ISO/IDF Standard (ISO, 2005) with slight modification as follows: 13g of freeze-dried yoghurt powder were reconstituted in 200g of distilled water at 24 °C, mixed well for 90 s using 3600 rpm, followed by centrifugation (6000 rpm for 5 min at 24 °C) in a graduated centrifuge tube. Volume per mL of sediment in the bottom of centrifuge tube was measured. Percentage of powder solubility was calculated using the following Eq. (4):

(4) $$\text{Solubility}\left(\text{\%}\right)=\frac{\text{Supernatant}\;\text{volume}}{\text{Total volume}}\times 100$$

### 2.12 Microstructure analysis, using scanning electron microscopy (SEM)

Morphological properties of freeze-dried yoghurts were carried out using scanning electron microscopy (SEM) at National Research Centre, Giza, Egypt. Samples of freeze-dried yoghurts were prepared as described by Jaya (2009) as follows: The sample was fixed on an iron stub and then made electrically conductive by coating it (in a vacuum chamber) with a thin layer of gold for 40 s. Moisture of freeze-dried samples was completely removed by placing the freeze-dried sample in an air-tight desiccator containing silica gel. The weight of samples was periodically measured until a constant weight to confirm complete removal of moisture. At least four images of typical structures at 1500 × magnification were recorded using a scanning electron microscope (FEI company, Netherlands) model quanta 250 FEG (field emission gun) attached with EDX unit (energy dispersive x-ray analyses), The images were taken at an excitation voltage of 20 K.V., at different magnifications varying from 400 to 6000 and working distance varying from 13.7-14.2 mm. Only 1500× magnification was shown for the present study.

### 2.13 Sensory evaluation

Sensory evaluation of fresh and rehydrated yoghurt types was performed after getting consent of the dairy science department's proving committee, Faculty of Agriculture, Benha University, Egypt. Sensory evaluation was done by 10 experienced panelists of staff members of the Dairy Department, Faculty of Agriculture, Benha University, after getting their consent. The evaluation – scheme III- approved by the American Dairy Science Association presented by Tamime and Robinson (2007). Flavour, body and texture, and appearance are the attributes used for evaluation. The score sheet was modified to be 45 points for flavor, 40 points for body and texture, and 15 points for appearance.

### 2.14 Statistical analysis

Statistical analysis was performed using analysis of variance (ANOVA) using the general linear models. Significant differences between samples mean of three replicates of data were determined at the (p < 0.05) levels.

## 3 Results and discussion

### 3.1 Physico-chemical properties of yoghurt

Data in Table 1 shows the physic-chemical properties of yoghurts as affected by treatments of fresh, freeze-dried, and rehydrated yoghurts. Fresh yoghurt was supplemented with 0.5% whey protein concentrate (WPC), modified starch (MS), or spirulina powder (SP) during its manufacturing. The physico-chemical properties of yoghurt were significantly affected by freeze drying and rehydration. The total solids, fat, protein, total carbohydrates, and ash contents were higher in rehydrated yoghurts than in fresh yoghurts. The total solids content in freeze-dried yoghurts were high and moisture content was less than 4.5% in the three types of freeze-dried yoghurts. Such values of moisture would lead to long shelf life of freeze-dried yoghurt powder Kearney et al., (2009), and Santos et al., (2018) Yoghurt acidity plays an important role in yoghurt flavor (Temesgen, 2015). All rehydrated yoghurt types had lower pH and higher acidity than those of fresh yoghurts due to the amount of water used for rehydration and the concentration effect during freeze drying process. These results are similar to those by Ibrahim (2018) and Santos et al. (2018). Supplementation of yoghurt with 0.5% WPC, MS, or SP, affected the chemical characteristics and increased the nutritional value of yoghurt. Addition of WPC or SP increased protein contents of fresh, freeze-dried, and rehydrated yoghurts. Addition of MS to yoghurt increased total sugars, and supplementation of yoghurt with WPC, SP, or MS increased the nutritional value of yoghurt, and protected the yoghurt starter cultures during freeze drying. Spirulina powder contains about 70% protein and it is a good source for essential fatty acids, amino acids, minerals, and vitamins, (Mosulishvili et al., 2004). Whey protein was added before freeze drying to protect the viability of yoghurt cultures during dehydration because it serves as protectants (Martín et al., 2015). Protein, fat, total carbohydrates, and ash contents were higher in the rehydrated yoghurts than in fresh yoghurts. The amount of water used for rehydration was less than the amount of removed water during freeze drying. These results led to more stable and nutritional yoghurt product as reported by Tamime and Robinson (2007).

**Table 1:** Physico-chemical properties of fresh, freeze-dried and rehydrated yoghurts (g∖100g).

Properties	Fresh yoghurt				Mean	Error Mean square	Freeze-dried yoghurt				Mean	Error Mean square	Rehydrated yoghurt				Mean	Error Mean square
	C1	W1	S1	M1			C2	W2	S2	M2			C3	W3	S3	M3		
TS	15.12^b^	15.31^a^	15.17^b^	15.20^ab^	15.2	0.007	95.75^a^	96.02a	95.86^a^	96.33^a^	95.99	0.138	26.59^c^	27.25^a^	26.85^b^	26.46^d^	26.79	0.0001
Fat	5.13^a^	5.05^b^	5.15^a^	5.03^b^	5.09	0.001	35.53^a^	33.96b	33.85^b^	34.46^b^	34.45	0.317	9.75^c^	9.95^a^	10.00^a^	9.85^b^	9.89	0.002
Protein	3.78^bc^	4.23^a^	4.03^ab^	3.62^c^	3.02	0.038	25.65^c^	27.35a	26.29^b^	25.44^c^	26.18	0.023	7.84^d^	8.76^a^	8.35^b^	8.01^c^	8.24	0.003
CHO	4.59^ab^	4.40^b^	4.33^b^	4.95^a^	4.57	0.67	24.62^b^	24.41b	24.80^b^	26.48^a^	25.08	0.653	5.59^a^	5.01^c^	5.14^bc^	5.27^b^	5.25	0.010
Ash	0.85^ab^	0.84^b^	0.84^b^	0.86^a^	0.85	0.0001	5.08^b^	5.22ab	5.35^a^	5.26^ab^	5.23	0.019	1.43^b^	1.66^a^	1.40^c^	1.37^d^	1.46	0.0001
Acidity	0.76^b^	0.78^b^	0.82^a^	0.75^c^	0.78	0.0001	4.87^bc^	5.08^b^	5.56^a^	4.70^c^	5.05	0.047	1.99^a^	1.88^c^	1.95^b^	1.96^b^	1.94	0.0001
pH	4.23^b^	4.28^b^	4.03^c^	4.34^a^	4.22	0.001	-	-	-	-	-	-	3.76^d^	3.99^a^	3.89^b^	3.85^c^	3.87	0.0001

**TS:** Total solids; **CHO**: Total carbohydrates**; C1:** fresh control sample; **C2**: Freeze-dried control sample; **C3**: rehydrated control sample; **W1:** fresh yoghurt containing whey protein isolate; **W2**: Freeze-dried yoghurt containing whey protein isolate; **W3**: rehydrated yoghurt containing whey protein isolate; **M1**: fresh yoghurt containing modified starch; **M2**: Freeze-dried yoghurt containing modified starch; **M3**: rehydrated yoghurt containing modified starch; **S1**: fresh yoghurt containing spirulina powder; **S2**: freeze-dried yoghurt containing spirulina powder; **S3**: rehydrated yoghurt containing spirulina powder. Different letters (a-d) in the same row indicate significant statistical differences (Duncan's test P < 0.05).

### 3.2 The texture profile of yoghurt

The texture profile of set-type yoghurt is important because of its vital role in consumers' acceptance. The texture profile parameters are shown in Table 2. Fracture, hardness, adhesiveness, cohesiveness, springiness, gumminess and chewiness explain the texture profiles of yoghurt. They affect the consumer's choice (Tamime and Robinson, 2007). The amount of water added to rehydrate yoghurts was 82% of the removed water by sublimation during the freeze drying. Santos et al. (2018) used 60, 65, and 70% of water sublimed during freeze drying process for rehydration, the reconstituted yoghurt reached about 75% of the weight of the traditional fresh yoghurt when 70% of water was used. Results show that fracture and hardness significantly increased in all rehydrated yoghurts. These results are due to the increase of total solids in rehydrated yoghurts. Addition of WPC, SP, or MS significantly decreased adhesiveness but significantly increased the cohesiveness, springiness, gumminess and chewiness in both fresh and rehydrated yoghurts, (Table 2). All rehydrated yoghurts needed penetration forces more than those needed for fresh yoghurts. This is due to presence of higher contents of total solids and lower contents of moisture in rehydrated yoghurts, where only 82% of sublimed water during freeze drying was added to rehydrate the freeze-dried yoghurt powder. Addition of 0.5% SP to yoghurt increased fracture, hardness, cohesiveness, springiness, gumminess, and chewiness in both fresh and rehydrated yoghurts as well as revealed the highest penetration force among all yoghurts. These results agree with those of Malik et al. (2013) who found that incorporation of 0.5 % spirulina increased penetration force indicating increase of strength of yoghurt curd. The increase of curd strength upon incorporation of spirulina could be attributed to the high protein content of spirulina. Consistency of yoghurt is important for acceptability of the consumers, an amount of water should be added to rehydrate the yoghurt powder to form a reconstituted yoghurt with texture profile that is similar to the traditional fresh yoghurt (Santos et al. (2018). Rehydration depends on the consumer’ preference. Increasing of water amount causes higher yield and lower viscosity of the reconstituted yoghurt. Rehydrated yoghurt with 82% of removed water during freeze drying caused a texture profile close to the traditional fresh yoghurt (Table 2). Venir et al., (2007) used 70% of removed water during freeze drying for rehydration of freeze-dried yoghurt. Akalın et al. (2012) observed that texture improvement of yoghurts upon many factors. Remeuf et al. (2003) found that WPC improved the texture and functional attributes of the yoghurt.

**Table 2:** Texture profile parameters of Fresh and rehydrated yoghurt.

Properties	Fresh yoghurt				Rehydrated yoghurt				Mean	Error Mean square	LSD
	C1	W1	S1	M1	C3	W3	S3	M3			
Fracture (N)	3.00^d^	2.25^e^	3.60^c^	2.40^e^	3.90^b^	3.70^bc^	4.25^a^	3.95^b^	3.381	0.023	0.265
Hardness (N)	3.45^d^	2.25^e^	3.60^cd^	2.40^e^	3.95^bC^	3.70^bcd^	4.35^a^	4.05^ab^	3.469	0.045	0.371
Adhesiveness (mj)	12.78^a^	1.48^c^	2.59^c^	1.16^c^	8.52^b^	0.85^c^	0.78^a^	0.77^c^	3.868	2.163	2.576
Cohesiveness (Ratio)	0.52^d^	0.61^ab^	0.66^a^	0.60^bc^	0.25^e^	0.57^bcd^	0.59^bc^	0.55^cd^	0.543	0.001	0.055
Springiness (mm)	7.45^c^	12.75^a^	12.75^a^	12.75^a^	9.07^b^	12.75^a^	12.75^a^	12.75^a^	11.626	0.144	0.664
Gumminess (N)	1.80^d^	1.40^e^	2.35^ab^	1.45^e^	1.00^f^	2.10^c^	2.55^a^	2.15^bc^	1.850	0.014	0.207
Chewiness (mj)	13.71^e^	17.47^d^	30.12^ab^	18.31^d^	8.92^f^	27.07^c^	32.13^a^	27.83^bc^	21.942	2.861	2.962

**C1:** fresh control sample; **C3**: rehydrated control sample; **W1:** fresh yoghurt containing whey protein isolate; **W3**: rehydrated yoghurt containing whey protein isolate; **M1**: fresh yoghurt containing modified starch; **M3**: rehydrated yoghurt containing modified starch; **S1**: fresh yoghurt containing spirulina powder; **S3**: rehydrated yoghurt containing spirulina powder. Different letters (**a-f**) in the same row indicate significant statistical differences (Duncan's test P < 0.05).

### 3.3 Evaluation of freeze-dried yoghurt powder

For evaluation of yoghurt powder, which was produced after freeze drying process, the related parameters are shown in Table 3. Yield of yoghurt powder produced after freeze drying ranged from 13.97% in the non-treated yoghurt to 16.69% in yoghurt containing WPC. The yield average after freeze drying was 15.07%. Average percentage of removed water from yoghurt after freeze drying was 84.93%. Santos et al. (2018) found that yield of yoghurt after freeze drying was 18% because they added 4% skim milk powder during manufacturing of traditional yoghurt.

**Table 3:** Removing water, yield, solubility and bulk density of Freeze-dried yoghurt.

Treatments	Removing water (%)	Yield (%)	Solubility %	Bulk density
C2	86.03^a^	13.97^b^	82.66^c^	1.3^b^
W2	84.30^b^	15.69^a^	84.35^b^	1.3^b^
S2	84.54^b^	15.46^a^	84.41^b^	2.0^a^
M2	84.84^b^	15.16^a^	86.01^a^	1.3^b^
Mean	84.929	15.071	84.358	1.475
Error Mean square	0.154	0.189	0.020	0.027

**C2**: Freeze-dried control sample; **W2**: Freeze-dried yoghurt containing whey protein isolate; **M2**: Freeze-dried yoghurt containing modified starch; **M3**: rehydrated yoghurt containing modified starch; **S2**: freeze-dried yoghurt containing spirulina powder; Different letters (**a-c**) in the same column indicate significant statistical differences (Duncan's test P < 0.05).

The solubility index of freeze-dried yoghurt was the highest in yoghurt containing MS where it recorded 86.01%, followed by 84.4 and 84.35% for yoghurts containing SP and WPC, respectively. The lowest solubility was 82.66% for the non-treated control yoghurt. It was noticeable that the addition of MS during yoghurt manufacturing significantly improved the solubility of freeze-dried yoghurt powder. Addition of SP during yoghurt manufacturing significantly increased the bulk density of freeze-dried yoghurt powder where, yoghurt containing SP revealed the highest bulk density (Table 3). The average bulk density of freeze-dried yoghurt powders was 1.475 g/mL. Akash and Yugal (2017) found that the average of bulk density of powder was 0.625 g/mL. Differences in bulk density is due to differences between milk and yoghurt structures. More bulk density occur in yoghurt powder than milk powder.

### 3.4 The water holding capacity (WHC) and syneresis

Water holding capacity (WHC) and syneresis of fresh and rehydrated yoghurts are shown in Table 4. WHC in all fresh yoghurts was higher than in rehydrated yoghurts. These results indicated that freeze-drying and water used in rehydration of yoghurt powder affected the WHC of rehydrated yoghurts. The WHC recorded low values in all rehydrated yoghurts. The decrease of WHC, upon freeze drying ranged from 22.12% in the control yoghurt to 29.9% in yoghurt containing SP. Addition of SP during yoghurt manufacturing significantly increased the WHC of fresh and rehydrated yoghurts. Akalın et al. (2012) found that whey protein concentrates enhanced water-holding capacity.

**Table 4:** Water holding capacity (WHC) and Syneresis of Fresh and rehydrated yoghurt.

Treatments	WHC (%)		WHC Decrease (%)	Syneresis (g/100g)	
	Fresh yoghurt	Rehydrated yoghurt		Fresh yoghurt	Rehydrated yoghurt
C	76.04^b^	59.22^a^	22.12	31.38^a^	NS
W	76.58^b^	54.64^d^	28.65	27.26^c^	NS
M	74.11^c^	56.19^c^	24.18	30.08^b^	NS
S	83.60^a^	58.60^b^	29.90	24.38^d^	NS
Mean	77.582	57.165	26.31	28.28	-
Error Mean square	0.215	0.0001	-	0.0001	-

**C:** control sample; **W:** yoghurt containing whey protein isolate; **M**: yoghurt containing modified starch; **S**: yoghurt containing spirulina powder. **NS**: No syneresis observed after 120min.; Different letters (**a-c**) in the same column indicate significant statistical differences (Duncan's test P < 0.05).

Syneresis of fresh yoghurt containing SP was the low where, it recorded 24.38g/100g. Differences between fresh yoghurts were significant. All rehydrated yoghurts showed no syneresis for 120 min. This is due to the high total solids content of all rehydrated yoghurts leading to more water trapping in the protein matrix of yoghurt. Yoghurts with high syneresis are low-quality products and the increase of total solids to 14% (wt/wt) decreased the syneresis. Addition of WPC did not increase syneresis. High WHC was obtained for yoghurts fortified with WPC (Akalın et al., 2012).

### 3.5 Viability of lactic acid bacteria

Viable counts of *Streptococcus thermophilus* in fresh yoghurts ranged from 6.97 to 7.02 log CFU/g, and in the rehydrated yoghurts ranged from 6.2 to 6.6 log CFU/g. *Streptococcus thermophilus* in fresh yoghurt containing MS was the highest. The viable counts of *Lactobacillus delbreuckii* subsp. *bulgaricus* in fresh yoghurts ranged from 7.74 to 7.80 log CFU/g. and in rehydrated yoghurts. Viable counts of *Lactobacillus delbreuckii* subsp. *bulgaricus* showed a slight increase where they ranged from 7.83 to 7.90 log CFU/g. These results indicated that freeze drying of yoghurt had minor effect on the viability *Streptococcus thermophilus* and had no effect on the viability of *Lactobacillus delbreuckii* subsp. *bulgaricus* and rehydrated yoghurts (Table 5). The current results are in a contrast with those found by Santos et al., 2018a, Santos et al., 2018b, who found that *Streptococcus thermophlius* usually survive more than *Lactobacillus bulgaricus* and that sucrose had no effect on protection of yoghurt starter culture during freeze drying. Whey protein was used as protectants and added to the drying media before freeze drying to protect the viability of yoghurt cultures during dehydration (Martín et al., 2015). Addition of sugar protected the yoghurt starter culture during freeze drying (Venir et al., 2007). Jaruwan Chutrtong (2015) found that the number of *L. bulgaricus* and *S. thermophilus* decreased 3 log cycles after freeze drying. Bacterial cells resistance to freeze drying depends on a variety of factors related to the bacterial cells and to the production conditions (Venir et al. (2007). The current results reveal that addition of WPC, MS or SP during yoghurt manufacturing serve as a protective agent for *Lactobacillus delbreuckii* subsp. *bulgaricus* from the effect of freeze drying. Ainaa et al. (2016) found that the use of guar gum, maltodextrin sucrose during freeze drying enhance the viability of the freeze-dried yoghurt. In a study of Venir et al. (2007) on freeze drying of three yoghurt types (skim milk yoghurt; whole milk yoghurt; yoghurt containing sucrose and blueberry), Lactobacilli and Streptococci counts decreased from 2.5 log CFU/g to 1.9 log CFU/g during freeze drying. Viability of total lactic acid bacteria in spirulina yoghurt was higher when compared to the other yoghurt types for both Lactobacilli and Streptococci. The current results are similar to those observed by Varga et al. (2002) and Malik et al. (2013) who found that spirulina had a positive effect on the survival of yoghurt starter bacteria. Spirulina have a positive effect on the survival of the lactic acid bacteria due to high contents of protein, vitamins, minerals and essential fatty acids especially gamma linolenic acid in spirulina (Perez et al., 2007).

**Table 5:** Microbiological examination of fresh and rehydrated yoghurt (Log cfu/g).

Starter culture	Fresh yoghurt				Rehydrated yoghurt			
	C1	W1	S1	M1	C3	W3	S3	M3
*Str. thermophilus*	6.97	7.00	7.01	7.02	6.60	6.50	6.53	6.20
*Lb. delbreuckii* subsp. *bulgaricus*	7.80	7.74	7.79	7.78	7.86	7.83	7.90	7.88
Total lactic acid bacteria	7.56	7.51	7.55	7.55	7.58	7.55	7.62	7.59

**C1:** fresh control sample; **C3**: rehydrated control sample; **W1:** fresh yoghurt containing whey protein isolate; **W3**: rehydrated yoghurt containing whey protein isolate; **M1**: fresh yoghurt containing modified starch; **M3**: rehydrated yoghurt containing modified starch; **S1**: fresh yoghurt containing spirulina powder; **S3**: rehydrated yoghurt containing spirulina powder.

### 3.6 Microstructure analysis

**Figure 1:**
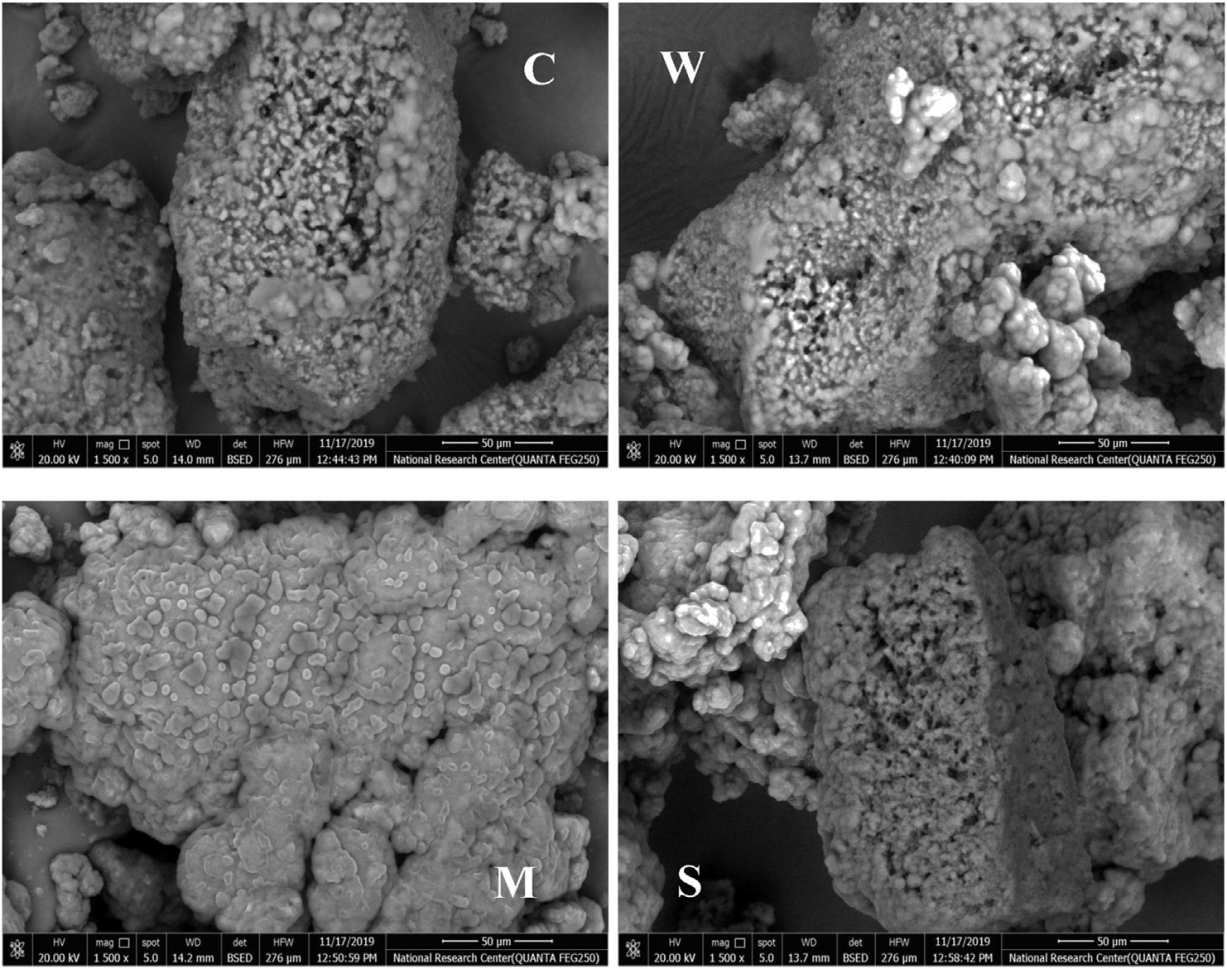
Scanning electron microscopy (SEM) images of freeze-dried yoghurts powders which were fortified with some additives during manufacture, (C): control sample; (W): yoghurt containing whey protein concentrate, (M): yoghurt containing modified starch; (S): yoghurt containing spirulina powder.

Scanning electron microscopy was used to investigate yoghurt microstructure. Results show that freeze-dried yoghurts differed because of addition of WPC, MS, or SP. Fortification with these additives increased the levels of total solids, especially proteins in the case of addition of WPC and SP. The increase of total solids and protein or carbohydrates caused the development of a gel network by cross-linking during fermentation of yoghurt. Images of freeze-dried powder of yoghurts which were fortified with WPC, MS, and SP are shown in Figure 1. Microstructure of freeze-dried yoghurts fortified with MS addition revealed an image which is totally different from the other images. Microstructure analysis indicated that granule structure of freeze-dried yoghurt fortified with MS was smoother and denser than those of the control samples. The control freeze-dried yoghurt appeared with granular, coarse, and rough outer surfaces. Control samples were of more heterogeneous and interspersed structure. However, yoghurt fortified with MS had smoother, systematically distributed casein and less porosity structure in the casein network. Lorenzen et al. (2002) and Khalifa and Ibrahim (2015) stated that cross-link formation between milk proteins cause emulsion stability. Addition of WPC caused different surface structure with some fine cross-linking among globular casein and the structure was porous. These structural features in addition to the cross-liking capacity of WPC would improve and reduce the syneresis due to increasing the bridging degree between protein particles. Puvanenthiran et al. (2002) and Akalın et al. (2012) observed that the protein network became finer and protein aggregates were smaller upon using whey proteins during yoghurt manufacturing. The microstructure of the freeze-dried yoghurt fortified with SP showed coarse and compact structure less porous in comparison with the freeze-dried yoghurts fortified with WPC. Addition spirulina powder increased total solids and proteins. High protein content affects the microstructure of the freeze-dried yoghurt fortified with SP where the surface appeared more compact. The use of some additives containing protein concentrates strongly affect the microstructure of yoghurts.

### 3.7 Sensory evaluation

Table 6 shows the sensory evaluation of yoghurts. Flavor of yoghurt containing WPC in both fresh and rehydrated types recorded the highest scores, where they were 42.75 and 43.00, respectively. Yoghurt, rehydrated from one-month old freeze-dried yoghurt powder kept at room temperature, had good properties (Jaruwan Chutrtong, 2015). The highest total acceptability scores were noted for yoghurt containing WPC, followed by the control yoghurt then yoghurt containing MS, and finally, yoghurt containing SP. Sensory evaluation results demonstrated that the rehydrated yoghurt had more acceptance than the fresh yoghurt, possibly due to the concentration of positively affected aroma and flavour compounds. The rheological behaviour of a reconstituted yoghurt powder was comparable with the fresh yoghurt since the yoghurt structural and rheological properties are closely related to the criteria of yoghurt sensory properties (as softness and consistency) which are directly related to consumer acceptance. Studies by Rathi (1990); Sakin-yilmazer et al. (2014) and Santos et al. (2018) showed that scores of appearance, flavor, taste, texture, and overall acceptance of reconstituted freeze-dried yoghurt are in line with results of current study.

**Table 6:** Sensory evaluation of Fresh and rehydrated yoghurt.

Properties (score)	Fresh yoghurt				Mean	Error Mean square	Rehydrated yoghurt				Mean	Error Mean square
	C1	W1	S1	M1			C3	W3	S3	M3		
Flavor (45)	41.50^b^	42.75^a^	40.50^c^	41.75^b^	41.63	0.188	42.0^ab^	43.0^a^	41.0^b^	42.0^ab^	42.00	0.83
Body & texture (40)	37.0^b^	38.0^a^	37.50^ab^	36.00^c^	37.13	0.063	37.0^ab^	38.0^a^	38.0^a^	36.0^b^	37.25	1.50
Appearance (15)	13.50^b^	14.5^a^	12.50^c^	13.75^b^	13.56	0.057	14.0^a^	14.0^a^	13.0^a^	14.0^a^	13.75	3.06
Total acceptability (100)	92.0^b^	95.25^a^	90.50^c^	91.50b^c^	92.31	0.307	93.0^a^	95.0^a^	92.0^a^	92.0^a^	93.00	4.28

**C1:** fresh control sample; **C3**: rehydrated control sample; **W1:** fresh yoghurt containing whey protein isolate; **W3**: rehydrated yoghurt containing whey protein isolate; **M1**: fresh yoghurt containing modified starch; **M3**: rehydrated yoghurt containing modified starch; **S1**: fresh yoghurt containing spirulina powder; **S3**: rehydrated yoghurt containing spirulina powder. Different letters (**a-c**) in the same row indicate significant statistical differences (Duncan's test P < 0.05).

## 4 Conclusion

Production of freeze-dried yoghurt powder fortified with whey protein concentrate (WPC), modified starch (MS), and spirulina (SP) could achieve yoghurt with considerable improved nutritional, textural, microbiological and sensory properties and can overcome the short shelf life of fresh yoghurt and cold storage. Fortification of yoghurt with WPC, MS, or SP can improve the sensorial characteristics, texture, and microstructure of yoghurt and protect the yoghurt starter microorganisms during freeze drying, especially in case of addition of MS. Addition of WPC caused improvement and decreased syneresis indices. Microstructure of freeze-dried yoghurt caused the structural changes The differences were a result of addition of WPC, MS, or SP. Addition of MS produced more smooth and less porous structure. Addition of SP produced more compact yoghurt. the present study suggests and concludes that freeze-dried yoghurt can be promising for marketing and that consumers will be willing to pay a higher price for such products.

## Declarations

### Author contribution statement

Elsayed A. Ismail: Conceived and designed the experiments; Performed the experiments; Analyzed and interpreted the data; Contributed reagents, materials, analysis tools or data; Wrote the paper.

Ahmed A. Aly: Performed the experiments; Analyzed and interpreted the data.

Atallah A. Atallah: Performed the experiments; Analyzed and interpreted the data; Contributed reagents, materials, analysis tools or data.

### Funding statement

This research did not receive any specific grant from funding agencies in the public, commercial, or not-for-profit sectors.

### Competing interest statement

The authors declare no conflict of interest.

### Additional information

No additional information is available for this paper.
